# Poultry hatcheries as potential reservoirs for antimicrobial-resistant *Escherichia coli*: A risk to public health and food safety

**DOI:** 10.1038/s41598-018-23962-7

**Published:** 2018-04-11

**Authors:** Kamelia M. Osman, Anthony D. Kappell, Mohamed Elhadidy, Fatma ElMougy, Wafaa A. Abd El-Ghany, Ahmed Orabi, Aymen S. Mubarak, Turki M. Dawoud, Hassan A. Hemeg, Ihab M. I. Moussa, Ashgan M. Hessain, Hend M. Y. Yousef

**Affiliations:** 1 Department of Microbiology, Faculty of Veterinary Medicine, Cairo University, Giza, Egypt; 2 Department of Civil, Construction and Environmental Engineering, Marquette University, Milwaukee, United States of America; 3 Department of Bacteriology, Mycology and Immunology, Faculty of Veterinary Medicine, Mansoura University, Mansoura, Egypt; 4 University of Science and Technology, Zewail City of Science and Technology, Giza, Egypt; 5 Department of Clinical and Chemical Pathology, Faculty of Medicine, Kasr AlAini, Cairo University, Giza, Egypt; 6 Department of Poultry Diseases, Faculty of Veterinary Medicine, Cairo University, Giza, Egypt; 7 Department of Botany and Microbiology, College of Science, King Saud University, Riyadh, Saudi Arabia; 8 Department of Clinical Laboratory sciences, college of Applied Medical sciences, Taibah University, Medina, Saudi Arabia; 9 Department of Health Science, College of Applied Studies and Community Service, King Saud University, Riyadh, Saudi Arabia; 10 Central Administration of Preventive Medicine, General Organization for Veterinary Service, Giza, Egypt

## Abstract

Hatcheries have the power to spread antimicrobial resistant (AMR) pathogens through the poultry value chain because of their central position in the poultry production chain. Currently, no information is available about the presence of AMR *Escherichia coli* strains and the antibiotic resistance genes (ARGs) they harbor within hatchezries. Therefore, this study aimed to investigate the possible involvement of hatcheries in harboring hemolytic AMR *E*. *coli*. Serotyping of the 65 isolated hemolytic *E*. *coli* revealed 15 serotypes with the ability to produce moderate biofilms, and shared susceptibility to cephradine and fosfomycin and resistance to spectinomycin. The most common β-lactam resistance gene was *bla*_TEM_, followed by *bla*_OXA-1_, *bla*_MOX_-like_,_ *bla*_CIT_-like_,_ *bla*_SHV_ and *bla*_FOX_. Hierarchical clustering of *E*. *coli* isolates based on their phenotypic and genotypic profiles revealed separation of the majority of isolates from hatchlings and the hatchery environments, suggesting that hatchling and environmental isolates may have different origins. The high frequency of β-lactam resistance genes in AMR *E*. *coli* from chick hatchlings indicates that hatcheries may be a reservoir of AMR *E*. *coli* and can be a major contributor to the increased environmental burden of ARGs posing an eminent threat to poultry and human health.

## Introduction

In poultry breeding, the hatchery occupies a central position between breeder farms and poultry production houses. However, because of their intensive production systems and the movement of the produced chicks across long distances, hatcheries can serve as a reservoir and source of pathogenic microorganisms. Some chicks are already infected with Gram-negative bacteria at hatching, which can lead to death from yolk sac infection or bacterial chondronecrosis with osteomyelitis^1^. The risk of illness through contamination of chickens with pathogenic *E*. *coli* should be a concern for all parties from farm to fork, as infection could occur through the environment, equipment, feed and drinking water, insufficient cleaning and disinfection, people and chickens themselves. A pathogen-free hatchery environment therefore plays a crucial role in preventing the spread of pathogens in the poultry value chain^2^.

Although it has been documented that chickens are the most significant carrier of food-poisoning microorganisms causing illness in humans^3^, their role in the dispersal of antimicrobial-resistant (AMR) pathogens and antibiotic resistance genes (ARGs) into the food chain has not been given due consideration.

The prevalence of AMR *E*. *coli* isolates has increased in low- to moderate-income countries such as Egypt, likely as the result of the very liberal and uncontrolled use of antibiotics, to the extent that it is becoming a threat to medical and veterinary treatment efficacy^4^. Except for anecdotal information, little information is available about the administration of antibiotics in chicken hatcheries in Egypt. Surveillance data from neglected sources such as hatcheries will be essential to gaining insight into the apparently augmented virulence of *E*. *coli* strains. Surveillance data can detect correlation with antibiotic use and resistance leading to better practices to assure human treatment efficacy.

The presence of strains producing extended-spectrum β-lactamases (ESBLs) or AmpC β-lactamases in food products is a cause for particular concern because these phenotypes are usually accompanied by a low susceptibility to other classes of antibiotics^5^. The distribution of *E*. *coli* producing ESBLs worldwide has increased over the past decades and the genes encoding ESBLs have further evolved^6^. Several studies have characterized producers of ESBLs and AmpC β-lactamases isolated from food-production broiler chickens by testing flocks at the farm level and fecal samples at later production steps as cited by Reich *et al*.^5^. However, very little is known about the prevalence and diversity of ESBLs within hatcheries and the effects on hatchling exposure and infection.

We assessed 10 geographically separate hatcheries from Egypt as reservoirs for AMR pathogenic *E*. *coli* that have the potential to spread through the food chain and the environment. *E*. *coli* isolated from hatchlings and the hatchery environment were examined for hemolytic ability, serotype, ability to form biofilm, and resistance to antibiotics. Furthermore, we assessed the prevalence of ESBL and AmpC β-lactamases genes in the isolates. The results show that AMR *E*. *coli* are prevalent in Egyptian hatcheries, and that there is a large variety in the resistance to antibiotics among these isolates. The isolates obtained from the hatchlings did not group with the isolates obtained from various sites within the hatchery, indicating hatchlings may become contaminated with *E*. *coli* primarily through contact with other hatchlings.

## Results

### Prevalence of pathogenic *E*. *coli* among Egyptian hatcheries

We obtained samples from 10 different hatcheries located within 40 km of cities in geographically separate regions in Egypt. We collected 45 samples from the meconium of day-old hatchlings from each hatchery, a total of 450 hatchling samples. Environmental samples taken from the hatcheries consisted of 3 samples from 8 different locations (Table 1) making a total of 24 samples per a hatchery or 240 total environmental samples. Each sample was examined for pathogenic *E*. *coli* by isolation on selective and differential media followed by confirmatory PCR amplification of the *uidA* gene. Isolates were considered pathogenic based on presence of hemolytic activity. Sixty-five pathogenic *E*. *coli* isolates were recovered – 30 from the day-old hatchling meconium samples and 35 from sites in the hatcheries (Table 1). No *E*. *coli* was detected in 3 of the 10 hatcheries analyzed and the remaining hatcheries showed presence of *E*. *coli* in both the hatchling meconium and hatchery locations. The positive relationship between presence of *E*. *coli* in the environment and the hatchling suggested that the hatchlings may have been exposed through its environment. To identify if the isolates from the environment were the same of similar as found in the hatchling, the isolates were further characterized.

**Table 1:** Number of *E*. *coli* isolates per matrix and hatchery.

Sampled Matrices	Hatcheries (H)										Total
	H1	H2	H3	H4	H5	H6	H7	H8	H9	H10	
Day-old hatchling meconium	4/45 (8.9%)	4/45 (8.9%)	4/45 (8.9%)	5/45 (11.1%)	3/45 (6.7%)	5/45 (11.1%)	5/45 (11.1%)	0/45 (0.0%)	0/45 (0.0%)	0/45 (0.0%)	30/450 (6.7%)
Air tunnels	0/3 (0.0%)	0/3 (0.0%)	0/3 (0.0%)	1/3 (33.3%)	0/3 (0.0%)	1/3 (33.3%)	1/3 (33.3%)	0/3 (0.0%)	0/3 (0.0%)	0/3 (0.0%)	3/30 (10.0%)
Incubators	0/3 (0.0%)	0/3 (0.0%)	3/3 (100%)	1/3 (33.3%)	1/3 (33.3%)	1/3 (33.3%)	1/3 (33.3%)	0/3 (0.0%)	0/3 (0.0%)	0/3 (0.0%)	7/30 (23.3%)
Hatchery machines	0/3 (0.0%)	0/3 (0.0%)	2/3 (66.6%)	1/3 (33.3%)	0/3 (0.0%)	1/3 (33.3%)	1/3 (33.3%)	0/3 (0.0%)	0/3 (0.0%)	0/3 (0.0%)	5/30 (16.7%)
Infertile eggs	2/3 (66.6%)	2/3 (66.6%)	0/3 (0.0%)	1/3 (33.3%)	0/3 (0.0%)	1/3 (33.3%)	1/3 (33.3%)	0/3 (0.0%)	0/3 (0.0%)	0/3 (0.0%)	7/30 (23.3%)
Water	0/3 (0.0%)	2/3 (66.6%)	1/3 (33.3%)	0/3 (0.0%)	1/3 (33.3%)	0/3 (0.0%)	0/3 (0.0%)	0/3 (0.0%)	0/3 (0.0%)	0/3 (0.0%)	4/30 (13.3%)
Workers’ hands	1/3 (33.3%)	1/3 (33.3%)	0/3 (0.0%)	0/3 (0.0%)	1/3 (33.3%)	0/3 (0.0%)	0/3 (0.0%)	0/3 (0.0%)	0/3 (0.0%)	0/3 (0.0%)	3/30 (10.0%)
Egg refrigerators	0/3 (0.0%)	1/3 (33.3%)	0/3 (0.0%)	1/3 (33.3%)	0/3 (0.0%)	1/3 (33.3%)	0/3 (0.0%)	0/3 (0.0%)	0/3 (0.0%)	0/3 (0.0%)	3/30 (10.0%)
Floors	0/3 (0.0%)	1/3 (33.3%)	0/3 (0.0%)	1/3 (33.3%)	0/3 (0.0%)	1/3 (33.3%)	0/3 (0.0%)	0/3 (0.0%)	0/3 (0.0%)	0/3 (0.0%)	3/30 (10.0%)
Total	7/69 (10.1%)	11/69 (15.9%)	10/69 (14.5%)	11/69 (15.9%)	6/69 (8.7%)	11/69 (15.9%)	9/69 (13.0%)	0/69 (0.0%)	0/69 (0.0%)	0/69 (0.0%)	

H1–H10 = Hatcheries 1 to 10; Hatchery samples were obtained from two visits. A total of 45 hatchling samples and 3 samples per environmental site were obtained per a hatchery.

Number of *E*. *coli* isolates/number of samples (percentage of isolates).

### Summary of the Characterization of *E*. *coli* isolates

To identify if the isolates from the environment are phenotypically the same or similar to those from the hatchling meconium, the isolates were examined for hemolytic ability, serotype, ability to form biofilm, and resistance to antibiotics. The *E*. *coli* isolates were serotyped to determine association with known pathogenic serotypes. Fifteen different *E*. *coli* serotypes were identified from different sources in the hatcheries, with O128:K71 the most prevalent serotype (Tables S1 and S2). The O128:K71 was the only serotype detected of isolates from the environment and hatchlings of the same hatchery (H1) suggesting little overlap between the environmental and hatchling isolates. Of the 65 isolates, 28 were identified with serotypes previously associated with isolates from chicken hatcheries or pathogenic strain serotypes O1, O2, O8, O78, O119, and O126.

Biofilm formation was measured to determine the ability of isolates to colonize surfaces for environmental survival and persistence and a virulence factor. The ability to form biofilm as determined by slime production (assessed by Congo red uptake and an adherence assay in glass tubes) revealed a heterogeneity among the isolates, ranging from weak and moderate to strong biofilm formation (Tables S1 and S2). The greater abundance of strong producers from the environmental isolates compared to the hatchling suggest that slim production may be associated with successful survival or colonization of hatchery surfaces (*p* = 0.009, Table 2).

**Table 2:** Phenotypes and genotypes identified as significantly different (*p* < 0.05) between the source of isolation (hatchlings *vs*. hatchery environments).

Phenotypes	Source	Prevalence (%)	*p*-value
Ciprofloxacin (R)	Hatchery environment	62.9	0.008
	Chicken hatchlings	30	
*bla*_SHV_ (+)	Hatchery environment	14.3	0.019
	Chicken hatchlings	40	
*bla*_*OXA*_ (+)	Hatchery environment	88.6	0.008
	Chicken hatchlings	60	
*bla*_*MOX*_-like (+)	Hatchery environment	11.4	<0.001
	Chicken hatchlings	90	
*bla*_FOX_ (+)	Hatchery environment	0	0.001
	Chicken hatchlings	26.7	
*bla*_CIT_-like (+)	Hatchery environment	8.6	<0.001
	Chicken hatchlings	66.7	
CR (+)	Hatchery environment	8.6	0.009
	Chicken hatchlings	36.7	
CR (+++)	Hatchery environment	31.4	0.009
	Chicken hatchlings	10	

The susceptibility of the 65 *E*. *coli* isolates to 23 antibiotics (Table 3) was evaluated (Tables S1 and S2) through disk diffusion method. All of the 65 *E*. *coli* isolates were susceptible to cephradine and fosfomycin and resistant to spectinomycin. All hatchling isolates demonstrated resistance to doxycycline and oxytetracycline, while sensitivity to colistin (Table S1). All environmental isolates showed sensitivity to gentamycin (Table S2). Additionally, the presence of ciprofloxacin resistance was significantly greater in the hatchery environmental isolates compared to the hatchling isolates (*p* = 0.008, Table 2). The different antibiotic resistances suggested that no isolate from the environment and hatchery were clonal, or of the exact same isolate.

**Table 3:** List, classification and prioritization of antimicrobials categorized as critically important in human and veterinary medicine.

Antibiotic	Disc concentration	Antimicrobial class	Medical importance (53)	Prioritization criterion
Colistin	600 µg	Polymyxins	Highest Priority Critically Important Antimicrobials	P1, P2 and P3
Cephradine	10 µg	Cephalosporins	Highly Important Antimicrobials	NA
Ceftiofur	10 µg	Cephalosporins	Highest Priority Critically Important Antimicrobials	P1, P2 and P3
Fosfomycin	5 µg	Phosphonic acid derivatives	High Priority Critically Important Antimicrobials	P1 and P2
Gentamycin	20 µg	Aminoglycosides	High Priority Critically Important Antimicrobials	P2 and P3
Neomycin	30 µg	Aminoglycosides	High Priority Critically Important Antimicrobials	P2 and P3
Streptomycin	5 µg	Aminoglycosides	High Priority Critically Important Antimicrobials	P2 and P3
Chloramphenicol	15 µg	Amphenicols	Highly Important Antimicrobials	NA
Enrofloxacin	10 µg	Quinolones and fluoroquinolones	Highest Priority Critically Important Antimicrobials	P1, P2 and P3
Ciprofloxacin	10 µg	Quinolones and fluoroquinolones	Highest Priority Critically Important Antimicrobials	P1, P2 and P3
Norfloxacin	10 µg	Quinolones and fluoroquinolones	Highest Priority Critically Important Antimicrobials	P1, P2 and P3
Flumequine	5 µg	Quinolones and fluoroquinolones	Highest Priority Critically Important Antimicrobials	P1, P2 and P3
Pefloxacin	5 µg	Quinolones and fluoroquinolones	Highest Priority Critically Important Antimicrobials	P1, P2 and P3
Amoxicillin	10 µg	Penicillins	Highest Priority Critically Important Antimicrobials	P2 and P3
Ampicillin	10 µg	Penicillins	Highest Priority Critically Important Antimicrobials	P2 and P3
Sulfamethoxazole/Trimethoprim	15 µg	Sulfonamides, dihydrofolate reductase inhibitors combination	Highly Important Antimicrobials	NA
Spiramycin	5 µg	Macrolides and ketolides	Highest Priority Critically Important Antimicrobials	P1, P2 and P3
Erythromycin	20 µg	Macrolides and ketolides	Highest Priority Critically Important Antimicrobials	P1, P2 and P3
Spectinomycin	5 µg	Aminocyclitols	Important Antimicrobials	NA
Rifampicin	5 µg	Ansamycins	Highest Priority Critically Important Antimicrobials	P1 and P2
Oxytetracyclin	20 µg	Tetracyclines	Highly Important Antimicrobials	NA
Doxycycline	20 µg	Tetracyclines	Highly Important Antimicrobials	NA
Clindamycin	20 µg	Lincosamides	Highly Important Antimicrobials	NA

**Prioritization criterion 1 (P1)**: High absolute number of people, or high proportion of use in patients with serious infections in health care settings affected by bacterial diseases for which the antimicrobial class is the sole or one of few alternatives for treating serious infections in humans. **Prioritization criterion 2 (P2)**: High frequency of use of the antimicrobial class for any indication in human medicine, or high proportion of use in patients with serious infections in health care settings, because use may favor selection of resistance in both settings. **Prioritization criterion 3 (P3)**: The antimicrobial class is used to treat infections in people for whom there is evidence of transmission of resistant bacteria (e.g., non-typhoidal *Salmonella* and *Campylobacter* spp.) or resistance genes (high for *E*. *coli* and *Enterococcus* spp.) from non-human sources. NA: not available.

As the spread of ESBL and β-lactam resistant bacteria are a human health concern, the presence of the ESBL class A β-lactamase genes *bla*_TEM_, *bla*_SHV_, *bla*_OXA-1_ and the *amp*C type β-lactamase genes *bla*_MOX_-like_,_ *bla*_CIT_ -like and *bla*_FOX_ were revealed through PCR analysis in 96.9%, 16.9%, 60.0%, 38.4%, 3.0%, and 27.7% of the *E*. *coli* isolates, respectively. These genes were chosen based on large variability in detection in *E*. *coli* adding a distinguishing variable and their known association with plasmids allowing horizontal gene transfer. The isolates from hatchlings were significantly greater in prevalence of all *ampC* type genes *bla*_MOX_-like, *bla*_CIT_ -like and *bla*_FOX_ by 78.6%, 58.1%, and 26.7%, respectively (*p* < 0.05, Table 2). The *bla*_FOX_ gene was not detected in isolates from the hatchery environment. The *bla*_SHV_ gene was also significantly greater in abundance in the hatchlings compared to the environment (*p* = 0.019). The *bla*_OXA-1_ gene was the only gene showing significantly greater abundance in the environment of the hatchery compared to the hatchlings (*p* = 0.008). This suggests that the genotype of the isolates from the hatchling and their environment related to the harboring of β-lactamases were different.

### Isolation, distribution, virulence, phenotypic features and antibiotic resistance traits of the *E*. *coli* isolates from hatchlings

Serotyping of the 30 *E*. *coli* isolates from the hatchlings revealed that they belonged to 10 different serotypes: O1:K61 (6.7%), O2:K69 (6.7%), O8:K60 (10.0%), O25:K- (6.7%), O78:K80 (10.0%), O86:K61 (20.0%), O119:K69 (10.0%), O128:K71 (13.3%), O158:K- (10.0%) and O164:K- (6.7%) (Table S1).

Hemolysin production was determined to differentiate between the virulent hemolytic isolates and the avirulent non-hemolytic isolates (Table S1). Out of the 30 hatchling isolates, 29 were capable of producing α-hemolysin; one serotype O86:K61 isolate produced β–hemolysin instead of α–hemolysin. Biofilm formation was measured to determine the ability of hatchling isolates to colonize surfaces for environmental survival and persistence and a virulence factor. Testing the ability to form biofilms, as determined by slime production (assessed by Congo red uptake and an adherence assay in glass tubes) revealed a heterogeneity among the hatchling isolates, ranging from weak and moderate to strong biofilm formation (Table S1). When screened for their adherence to polyvinyl chloride (PVC) 96-well microtiter plates, a moderate ability to form biofilms was observed for all hatchling isolates (OD_595_ < 1).

The results of the antibiotic resistance profiles for the 30 *E*. *coli* isolates from hatchling meconium (Table S1) showed β-lactam antibiotics tested were inactive against many of these isolates, with 0.0%, 90.0%, 90.0% and 56.7% of isolates resistant to cephradine, amoxicillin, ampicillin and ceftiofur, respectively. Norfloxacin was the least active (93.3% resistant isolates) of the fluoroquinolone (FQ) antimicrobials, with flumequine, another FQ, yielding similar results (90.0% resistant isolates) and resistance to pefloxacin (80.0% resistant isolates) and enrofloxacin (60.0% resistant isolates) also common. In contrast, ciprofloxacin was quite active, with resistance in only 30.0% of hatchling isolates. No resistance was detected against colistin and fosfomycin, 10.0% were resistant to gentamycin. Most of the isolates were resistant to most of the remaining antibiotics; in descending order, all were resistant to doxycycline, oxytetracycline and spectinomycin, 96.7% were resistant to clindamycin and erythromycin, 93.3% were resistant to streptomycin, 86.7% were resistant to rifampicin and spiramycin, 76.7% were resistant to sulfamethoxazole-trimethoprim drug combination, 73.3% were resistant to neomycin and 53.3% were resistant to chloramphenicol. The high abundance of antibiotic resistant hatchling isolates, some last resort antibiotics for clinical treatment of human infection, suggest a reservoir of AMR *E*. *coli* and potential to disseminate.

Investigation of the class A β-lactamase genes encoding ESBLs, *bla*_TEM_, *bla*_SHV_, and *bla*_OXA-1_, revealed that these genes were present in 93.3%, 40.0% and 60.0% hatchling isolates, respectively. The *amp*C genes *bla*_FOX_, *bla*_MOX_-like and *bla*_CIT_-like were identified in 90.0%, 26.7% and 66.7% of the hatchling isolates, respectively. The distribution of the resistance genes varied between different hatchling isolates (Table S1). Interestingly, only 6.7% isolates from hatchlings harbored none of the 6 resistance genes, 10% of the isolates harbored 5 of the resistance genes (O78:K80, O86:K61, O128:K71) and 16.7% isolates carried all 6 resistance genes tested (all from serotype O86:K61). The prevalence of these β-lactamase genes and their close association with elements of horizontal gene transfer, plasmid, suggest the possibility of further dissemination of β-lactam resistance.

According to the definition of Souli *et al*.^7^, none of the 30 hatchling isolates were pan drug resistant (PDR), however, 63.3% isolates were multiple drug resistant (MDR; excluding XDR) and 36.7% were extensively drug resistant (XDR). These XDR *E*. *coli* isolates were resistant to 11 of the 13 classes of antibiotics tested and included three serotype O78:K80, two O128:K71, two O119:K58, two O164:K-, and two O2:K69. Nineteen different MDR and XDR patterns were observed. Interestingly, all serotype O158:K- hatchling isolates had the same resistance profile, showing resistance to 14 antibiotics, whereas 50% serotype O86:K61 isolates were resistant to 17 antibiotics. In addition, 50% serotype O128:K71 isolates shared a common resistance profile, showing resistance to the following 12 antibiotics while 66.7% serotype O8:K60 isolates were resistant to 15 antibiotics. The abundance of MDR and XDR within the hatchling isolates demonstrated a high presence of AMR *E*. *coli* that may eventually enter the poultry production chain.

### Isolation, distribution, virulence, phenotypic features and antibiotic resistance traits of the *E*. *coli* isolated from hatchery environments

Serotyping of the 35 *E*. *coli* isolated from hatchery environments revealed seven different serotypes (Table S2).

Investigation of the production of hemolysin by the 35 *E*. *coli* isolated from the hatchery environment revealed that 91.4% could produce α-hemolysin, whereas 8.6% isolates, from serotype O126:K71 (isolated from an incubator), O128:K71 (isolated from an infertile egg) and O114:K90 (isolated from water), produced β–hemolysin instead. Analysis of biofilm formation using Congo red uptake and glass test tube assays identified 17.1% hatchery isolates as weak biofilm formers, 54.3% as moderate biofilm formers and 28.6% as strong biofilm formers (Table S2), while all hatchery isolates had a moderate ability to form biofilms on polyvinyl chloride (PVC) (OD_595_ < 1), as indicated by adherence assays conducted in 96-well PVC microtiter plates.

Antibiotic resistance profiling of the 35 *E*. *coli* isolates from hatcheries showed that the β-lactam antibiotics tested were inactive against most of the isolates; all hatchery isolates were sensitive to cephradine, and resistance against amoxicillin and ampicillin was detected in 91.4% hatchery isolates for each antibiotic. Ceftiofur was the most active β-lactam antibiotic with 71.4% of hatchery isolates showing resistance. Among the FQ, pefloxacin was the least active, with 94.3% hatchery isolates displaying resistance. Ciprofloxacin was the most active FQ, with only 62.9% of the hatchery isolates showing resistance. The resistance for chloramphenicol was 42.9% of hatchery isolates. All hatchery isolates were sensitive to fosfomycin and gentamycin. All hatchery isolates were resistant to spectinomycin. Resistance for doxycycline, oxytetracycline, clindamycin and erythromycin was detected in 97.1% of isolates for each antibiotic. For streptomycin, spiramycin, and sulfamethoxazole-trimethoprim, 91.4% resistant isolates were detected, whereas 85.7% isolates were resistant to rifampicin and 80.0% to neomycin. The high abundance of antibiotic resistant isolates from the hatchery environment suggest a reservoir of AMR *E*. *coli* and potential infection or dissemination to hatchlings.

The occurrence of class A β-lactamase genes and *amp*C genes assessed by PCR (Table S2) indicated that their distribution varied in the hatchery isolates. The *bla*_TEM_, *bla*_SHV_, and *bla*_OXA-1_ genes were identified in 97.1%, 14.3% and 88.6% of the hatchery isolates, respectively, whereas the *amp*C genes *bla*_FOX_, *bla*_MOX_-like, and *bla*_CIT_-like were identified in 0%, 11.4% and 8.6% of the hatchery isolates, respectively. Three isolates contained only one of the six resistance genes, 22 isolates contained two resistance genes and 10 isolates contained three resistance genes. We identified 7 isolates that produced an ESBL that also carried an *ampC* gene. The high abundance of ESBL related genes *bla*_TEM_ and *bla*_OXA-1_ indicate that the hatchery environment is a reservoir of these genes and may potentially disseminate through the poultry production change through hatchlings.

#### Multiple resistance patterns and distribution among *E. coli* serotypes

Of the 35 *E*. *coli* hatchery isolates, none were PDR, 22.9% were XDR, and 77.1% of isolates were MDR (excluding XDR; Table S2). Of the XDR isolates, one serotype O78:K80 and one serotype O119:K69 isolate were resistant to 12 of the 13 antibiotic classes tested. The other XDR isolates were one O119:K69, one O128:K71, one O119:K69, two serotype O128:K71, one O126:K71 serotype isolate were to 12 classes of antibiotics tested. There was 32 different MDR and XDR patterns observed, and interestingly 30 of them were unique to a single *E*. *coli* hatchery isolate. The diversity of resistance patterns suggest a potentially diverse pool of resistance genes or associations of genes such a plasmid within the hatchery environment.

### Associations between serotype, isolation source and phenotypic and genotypic traits

**Figure 1:**
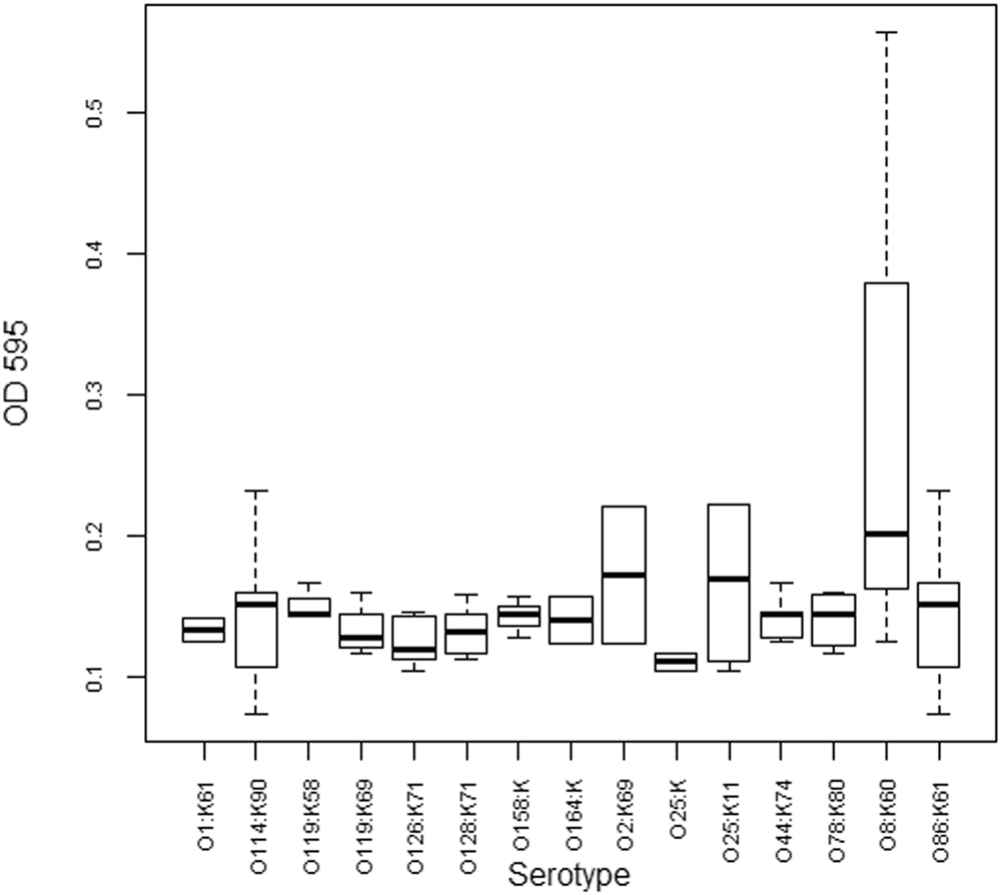
Biofilm formation in the microtiter plate assay by serotype.

**Figure 2:**
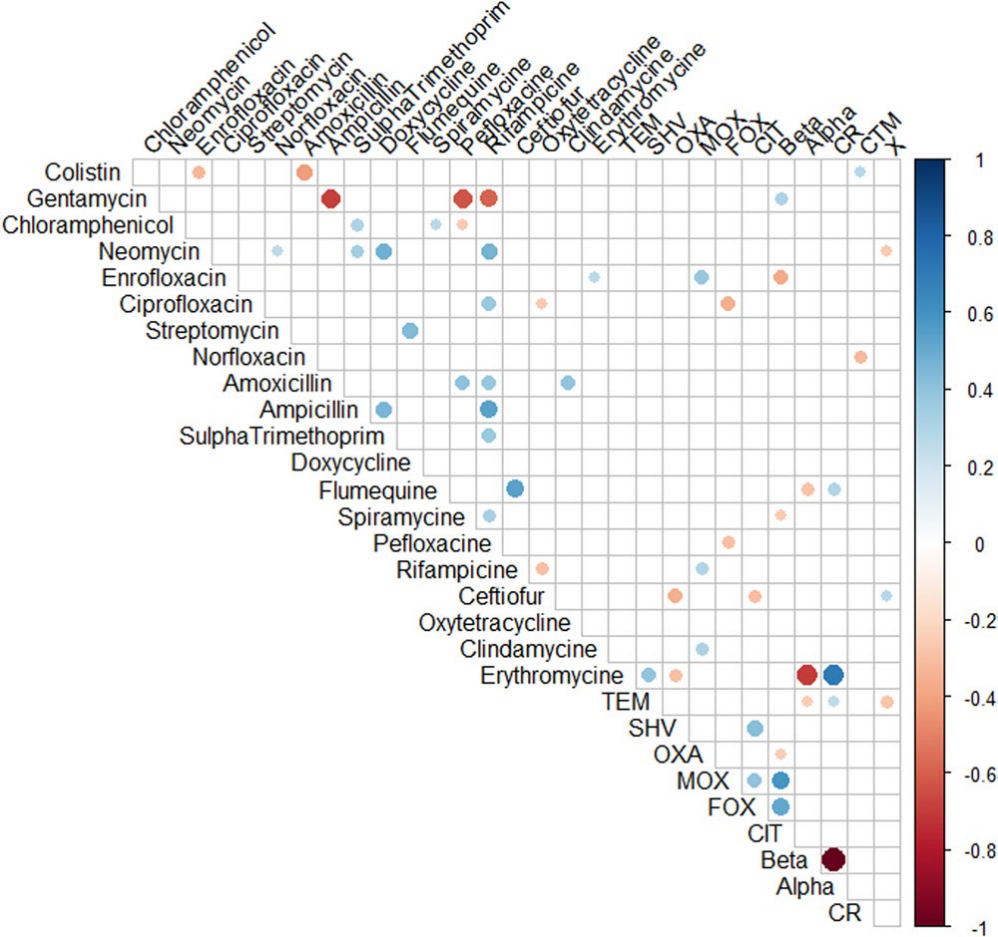
Correlation matrix of phenotypic (antibiotic resistance, hemolytic activity and biofilm formation ability) and genotypic (antibiotic resistance genes) features showing significant (p < 0.05) correlations. White spaces are not significantly correlated. Blue circles indicated significant positive correlation and red show significant negative correlation. The size and strength of color represent the numerical value of the Phi correlation coefficient.

The relationship of the strength of biofilm formation and serotype was performed to determine possible associations in the isolates. Serotype may have a relationship with biofilm forming ability in PVC microtiter plates, with serotype O8:K60 isolates being stronger biofilm formers and serotype O25:K- isolates forming the weakest biofilms (Fig. 1), however, more isolates are needed to demonstrate this relationship with any statistical significance. There were no significant differences between serotypes in the formation of biofilm as determined by the CTM and MTP methods (*p* = 0.30 and 0.31 respectively). While statistical analysis suggested a possible significant difference in serotypes impact on biofilm formation determined by CR (*p* = 0.013) there was no significant difference upon pairwise comparisons (*p* > 0.171) there was also no significant correlation between CTM and CR results (*p* > 0.05, Fig. 2).

**Figure 3:**
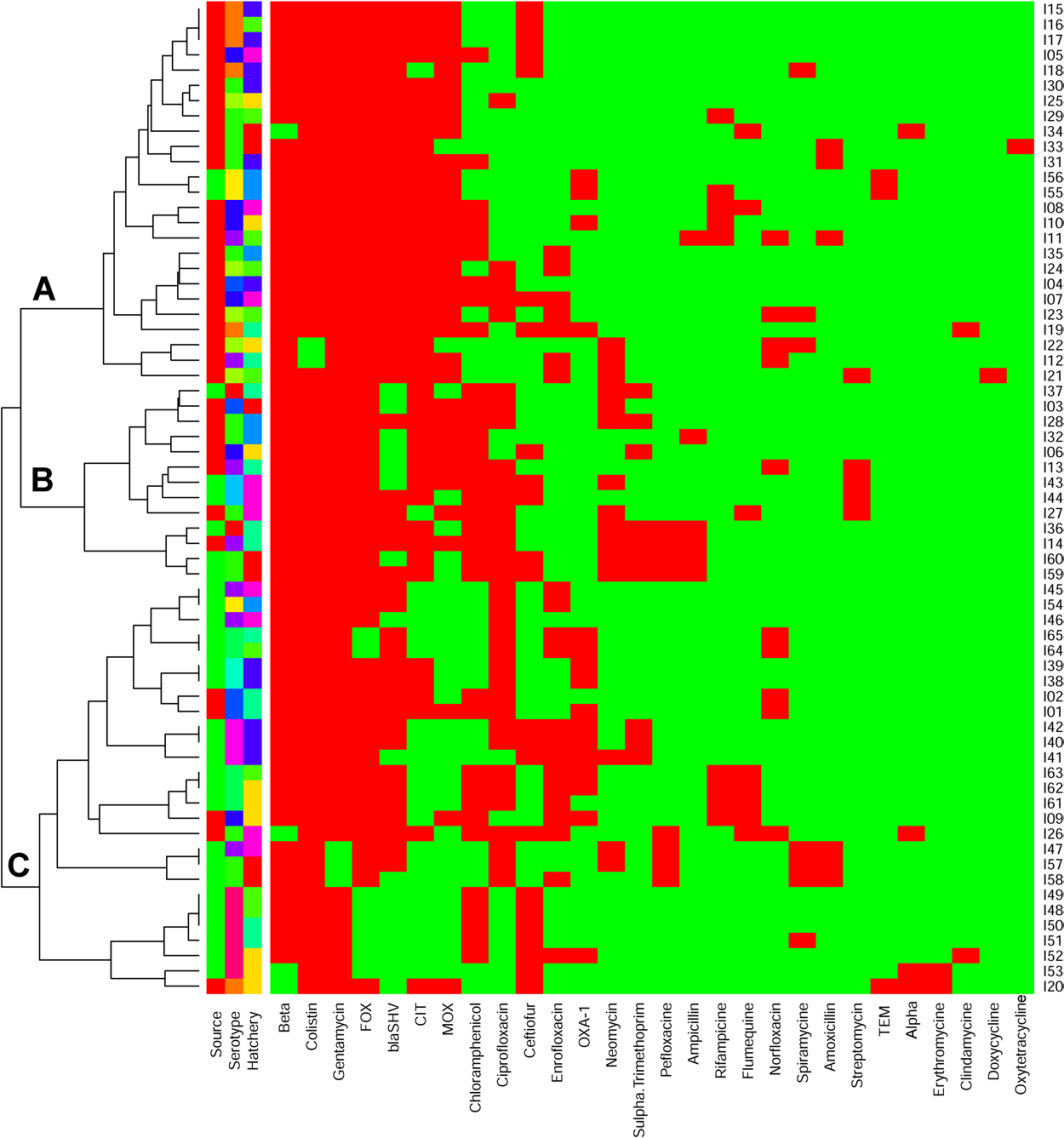
Heatmap and hierarchical clustering of *E*. *coli* isolates according to their phenotypic (antibiotic resistance) and genotypic (antibiotic resistance genes) profile of variables showing differences between isolates. Red represent presence and green represented absence of resistance or gene. Left of the heatmap is color representation of the different sources (hatchling in green and hatchery in red), the different serotypes, and the different hatcheries. Hierarchical clustering was perform using Wald’s method and a binary distance matrix. Letters designate the 3 main clusters described in the text.

**Figure 4:**
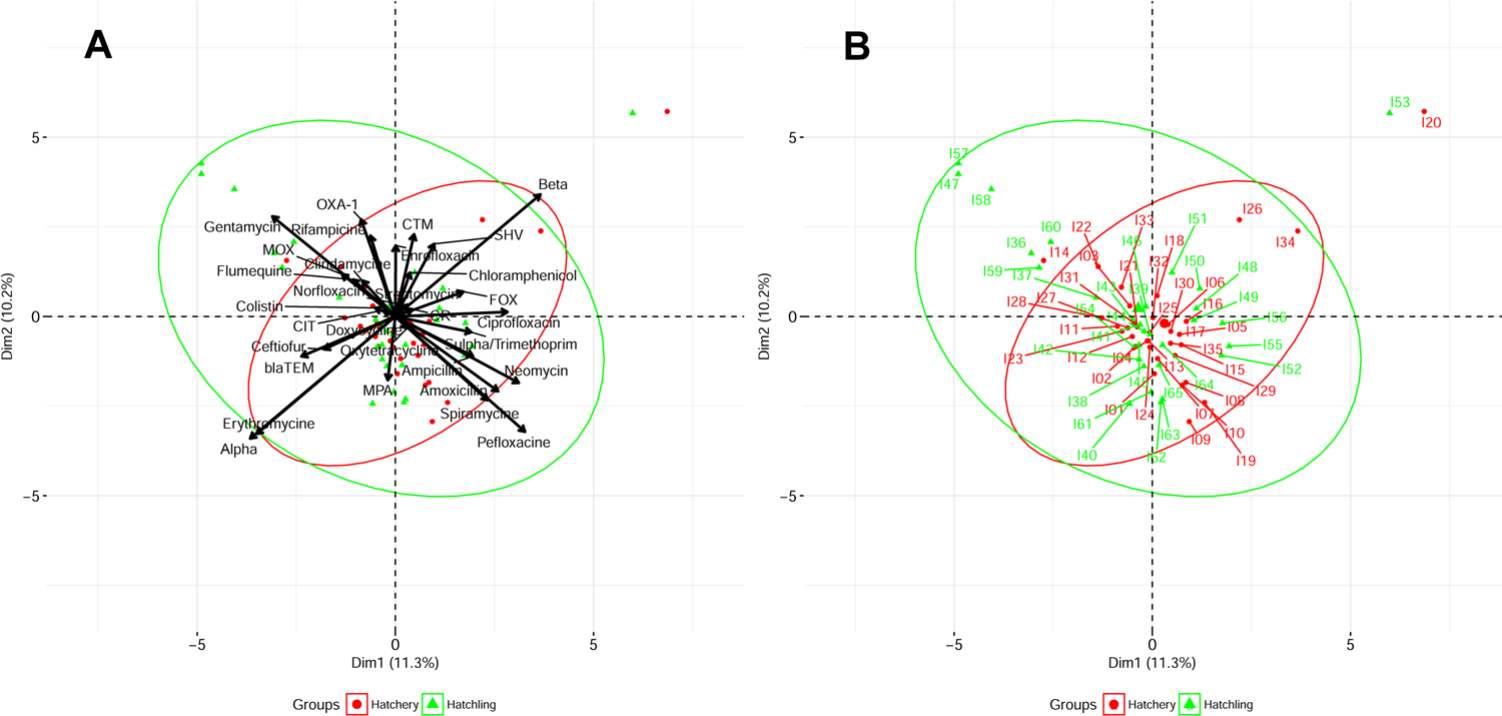
Principle component analysis performed on variables showing differences. (**A**) Visualization of the isolates encompassed in 95% confidence intervals grouping based on source of the isolate (hatchery or hatchling) and (**B**) labeling of the individual isolates from the same analysis.

Correlation matrix analysis (Fig. 2), hierarchical clustering (with heatmap) (Fig. 3), principle component analysis (Fig. 4) was used to determine associations between the phenotypic and genotypic traits and source of the isolates. Correlation analysis showed very few positive relationships with the presence of β-lactamase genes and resistance to β-lactams except for resistance to ceftiofur correlating with the presence of the *bla*_SHV_ gene (Fig. 3, *p* < 0.05). Significant positive correlations of antibiotic resistances indicated co-occurrence of resistance may be prevalent (*p* < 0.05, Fig. 3) and confirmed the presence of MDR and XDR strains (above). For example, resistance to the β-lactam amoxicillin was positively correlated with resistance to spiramycine, pefloxacine, and oxytetracycline (*p* < 0.05). Similarly, resistance to the β-lactam ampicillin was positively correlated with resistance to combination drug sulfamethoxazole-trimethoprim, spiramycine, chloramphenicol, and neomycin (*p* < 0.05). The Principle Component Analysis (PCA) showed similar relations between these correlations with β-lactam resistance (Fig. 4). However, resistance to the β-lactam ceftiofur did not show any positive correlations with any other antibiotic resistances tested. The presence of *amp*C related β-lactamase genes of *bla*_MOX_-like and *bla*_CIT_-like were positively correlated with quinolone resistance to enrofloxacin, ciprofloxacin, or perofloxacine (*p* < 0.05) also visualized in the PCA.

Hierarchical clustering analysis allowed the segregation of the 65 *E*. *coli* isolates according to their phenotypic (antibiotic resistance profiles, biofilm formation ability, hemolysis) and genotypic (β-lactam resistance gene profiles) traits (Fig. 3). Interestingly, a separation of *E*. *coli* isolated from chick hatchlings (Fig. 3, Cluster A) and *E*. *coli* isolated from hatchery environmental samples (Fig. 3, Cluster C) was observed. Three main clusters were identified. Cluster A included 23 *E*. *coli* isolates from chick hatchlings (from serotypes O128:K71, O78:K80, O158:K-, O86:K61, O164:K-, O8:K60, O119:K58 and O2:K69) and 2 from environmental isolates from water of the same hatchery (serotype O114:K90 and O44:K74), Another large cluster (Cluster C) contained 25 *E*. *coli* isolates from the hatchery environment (serotypes: O119:K69, O78:80, O114:K90, O25:K11, O44:K74, O128:K71, and O126:K71) and 2 isolates from hatchlings from the same hatchery (serotype: O119:K58). The third cluster (Cluster B) consisted of 7 hatchling isolates and 6 environmental isolates. Of interest in the third cluster, a hatchling isolate (serotype O78:K80, Isolate 27) and environmental isolate from egg refrigerator (serotype 01:K61, Isolate 44) from the same hatchery were clustered together. While being of different serotypes, these two isolates demonstrated the same phenotype and genotype. The hatchling isolates 36, 59, and 60 clustered together with the hatchery isolate 14 in PCA (Fig. 4) and hierarchical clustering (Fig. 3) most likely driven by the sensitivity to ampicillin, sulfamethoxazole-trimethoprim, and pefloxacine among other shared similarities. Similarly, the clustering of hatchling isolates 47, 57, and 58 were visible in both the PCA and hierarchical clustering and was driven by the presence of gentamycin resistance, as other isolates lacked this property. PCA showed that the hatchery isolates 20 and hatchling isolate 53 clustered far from the majority of isolated and shared the same antibiotic resistances to including sensitivity to erythromycin, which all other isolates showed resistance. There is no evidence that any two isolates had the same phenotype therefore are non-clonal indicating that there was no demonstration of direct transfer between hatchery environment and hatchling, or vis-versa. The differences in associations of traits as demonstrated by clustering, PCA, and correlation indicate the potential for hatchlings and hatchery environment to select for different traits within *E*. *coli*.

## Discussion

Not only is there a limited amount of information in the literature regarding the prevalence of *E*. *coli* and antibiotic resistance in hatcheries, but there is also a considerable variation in those reports^8^. Several factors may have contributed to these very different estimates of *E*. *coli* prevalence. The methods used for obtaining the samples from the hatcheries were not consistent across the different studies. The potential effect of the differences in methodology between studies, such as in the media used for enrichment, selective enrichment, and isolation, can also affect estimates of prevalence^9^.

The serotyping of the 65 isolates in our study revealed several interesting aspects of the isolates. Among the isolates in Egyptian hatcheries, we identified pathogenic *E*. *coli* serogroups, such as O1, O2, and O78, that are usually implicated in field infections^10^. Furthermore, three of the 65 *E*. *coli* isolates belonged to the O8 serogroup, which has been associated with hatchery losses and early chick mortality in India^11^, while *E*. *coli* serotypes O119 and O126, which were also serologically typed from hatchery isolates in Saudi Arabia, were also isolated in the present investigation^12^. Our sampled hatcheries were geographically separate and situated in a region with a high density of food animal production, which could indicate a common source or selective pressure, while the fertile eggs were supplied by different companies. Fortunately, the six non-O157 Shiga toxin-producing *E*. *coli* (STEC) serogroups that increasingly have been associated with serious outbreaks of human infection and often referred to as “the big six”^13^, O26, O45, O103, O111, O121, and O145, were not identified in our survey.

An important microbial characteristic associated with virulent avian *E*. *coli* is a hemolytic reaction on blood agar plates^14,15^ – hemolytic strains are more virulent than non-hemolytic strains^16,17^. α-Hemolysin, also known as cytotoxic necrotizing factor, is produced by invasive strains of *E*. *coli*, which sets the pace for the pathogenesis of renal disease^18^ and enhances virulence in a number of clinical infections^19^. The high prevalence of α-hemolysin in our isolates indicates that it is a common exotoxin produced by avian pathogenic *E*. *coli* strains. The ciprofloxacin- and FQ-resistant isolates show a lower expression of α-hemolysin and instead produced more β-hemolysin^20^.

The biofilm-producing abilities of the *E*. *coli* from the different other sources in the hatchery were monitored in our study using the CR assay, which is an easy and reproducible standard laboratory method. It has been used to obtain experimental evidence of amyloid formation and for the presence of a biofilm matrix^21^ and has previously been used to identify potentially pathogenic *E*. *coli* strains in hatcheries and brooders^22^. Several studies have indicated that only pathogenic *E*. *coli* can bind this dye and that Congo red-positive *E*. *coli* strains will cause disease when inoculated into chicks^23^. Little has previously been reported on potential differences in biofilm production between serotypes or serogroups of *E*. *coli*^24^, although differences between strains have been reported by several reports^25–27^. In our study, minor differences in the ability to form biofilm were observed among *E*. *coli* serotypes, although strains of serotype O8:K60 formed stronger biofilms on PVC. Nevertheless, strains of environmental origin showed a greater ability to form biofilms than *E*. *coli* strains isolated from chick hatchlings, which suggests that biofilm formation is important for environmental survival and persistence. *E*. *coli* biofilms can cause serious problems in hatcheries by increasing the resistance of cells to environmental stresses and protecting them from cleaning and sanitation procedures used to decontaminate processing environments^28^. To the best of our knowledge, no published study has compared biofilm production by poultry-related *E*. *coli* isolates with their zoonotic potential. It is conceivable that biofilms could allow bacteria to persist and survive in the hatchery on poultry processing equipment that is mostly made of glass and/or PVC material^29,30^ and thus can be colonized by pathogenic *E*. *coli* strains.

Antibiotic use plays a major role in the emerging public health crisis of antibiotic resistance, which has become a looming problem^31^. The majority of antibiotic use occurs in agricultural settings^31^, and agricultural antibiotics are associated with clinical antibiotic resistance^31^. Indeed, recently it was estimated that annually more than 1500 deaths in the European Union are directly related to antibiotic use in poultry^32^ and that 56% of the resistance genes in human pathogens were identical to genes derived from *E*. *coli* strains isolated from poultry sources^32^. Globally, billions of chickens receive third-generation cephalosporins, *in ovo* or as day-old chicks, to treat *E*. *coli* infection, a practice that has resulted in large reservoirs of resistant bacteria^32^. In our study, we show that resistance to antimicrobial classes commonly employed in industrial farming, including macrolides (erythromycin), tetracyclines (tetracycline) and penicillins (penicillin and ampicillin), is more prevalent than resistance to antibiotics that are less frequently used. The exception was lincosamide (clindamycin) resistance, which was relatively, and unexpectedly, low compared with resistance against other antimicrobial classes commonly used in animal rearing. An interesting finding of our study is the low resistance level observed for “the older antibiotics”, i.e., colistin, fosfomycin and cephradine, which are listed on the World Health Organization’s List of Essential Medicines and are considered among the most important drugs needed in a basic health system (Table 3)^33^. Colistin remains one of the antibiotics of last resort against MDR Enterobacteriaceae that produce the New Delhi *metallo*-β-lactamase (NDM-1)^34^ and it remains the only available treatment for XDR infections. None of the isolates were resistant to fosfomycin, a phosphonic acid derivative that has been used for the prophylaxis and treatment of UTI and has activity against ESBL-producing *E*. *coli*. Fosfomycin has therefore retained medical importance, either alone or in combination with other antimicrobials, including β-lactams, aminoglycosides, and fluoroquinolones, possibly owing to synergistic effects. In contrast, resistance to 3^rd^-generation cephalosporins such as ceftiofur was frequent in the hatcheries. Furthermore, we found that resistance to chloramphenicol and tetracycline often co-occurred. Resistance to these antibiotics is often linked to isolates carrying *bla*_SHV_ alone or in combination with resistance to nalidixic acid^5^. The co-resistance and the rate of reduced susceptibility found in our study are comparable to findings reported by Dierikx *et al*.^35^ and may be caused by treatment regimens similar to those used in conventionally reared broilers in Germany and the Netherlands.

Several *E*. *coli* isolates displayed MDR and XDR phenotypes. Correlation analyses showed that co-occurrence of resistance to various antibiotics, as has also been previously described^36,37^, represents an important concern for human and animal medicine alike. On the other hand, resistance to certain antibiotics was associated with susceptibility to others. For example, resistance to gentamycin appeared to be associated with susceptibility to amoxicillin, pefloxacin and spiramycin. This result is remarkable because it can facilitate the selection of alternative antibiotics when addressing MDR or XDR *E*. *coli* strains.

The detection of resistance genes in our investigation highlights the fact that *E*. *coli* found in poultry may serve as reservoirs for anti-microbial resistance genes that could potentially be transferred to pathogenic microorganisms infecting humans^38^. In the current study, the presence of three ESBL-encoding genes (*bla*_TEM_, *bla*_SHV_, and *bla*_OXA-1_) and three *ampC*-related β–lactamase genes (*bla*_MOX_-like_,_ *bla*_CIT_-like and *bla*_FOX_) in the *E*. *coli* isolates was determined. TEM-52, SHV-12, and CTX-M-1 are the most frequently reported ESBL types from the food animal reservoir^5,39,40^. We found that several antibiotic-resistant isolates lacked the *ampC*-related genes, indicating that these isolates might encode a different mechanism of resistance, potentially overexpression of chromosomal *amp*C, which usually results from mutations in the promoter/attenuator region of *amp*C^5,41^.

Interestingly, although no clear associations could be observed between serotype and antibiotic resistance phenotype or gene profile, isolates from chick hatchlings were more likely to contain the *bla*_SHV_, *bla*_MOX_-like_,_ *bla*_CIT_-like and *bla*_FOX_ resistance genes than isolates of environmental origin, which suggests that antibiotic use in poultry production is imposing a selective pressure, leading to the spread of these AMR genes in poultry hosts. However, no significant differences in resistance to the tested antibiotics were observed between isolates of animal and environmental origin, with the exception of ciprofloxacin, as ciprofloxacin resistance was higher in environmental isolates than in isolates of animal origin. Hierarchical clustering of *E*. *coli* isolates based on their phenotypic and genotypic profiles allowed for a good separation of isolates from hatchlings (which had a higher prevalence of β-lactamase encoding genes) and those from hatchery environments (which were more resistant to ciprofloxacin and had better abilities to form biofilms), which suggests that isolates from hatchlings and environmental isolates have different origins. The main differences among isolates of animal and environmental origin potentially reflect the different lifestyle of the pathogen in both niches, in that isolates of animal origin are more frequent carriers of *ampC* genes, likely due to the increased selective pressure imposed by the use of antibiotics in farms. Moreover, isolates of environmental origin were stronger biofilm formers, likely because the biofilm lifestyle helps them persist in the hatchery environment.

The lack of uniform surveillance protocols for pathogenic *E*. *coli* and antimicrobial resistance genes at hatcheries constitutes an important gap in the farm-to-fork spectrum of food safety protection and a risk to public health and food safety that needs to be addressed. The findings described in this study are significant to public health because they indicate that the potential benefit of post-Hazard Analysis and Critical Control Point reduction in pathogenic *E*. *coli* prevalence at processing plants can be undermined by contamination occurring at the hatchery. Moreover, we have found that resistance to β-lactams and other clinically relevant antibiotics is widespread in hatcheries, including the presence of several MDR and XDR isolates.

## Materials and Methods

### Study area

Ten hatcheries located in the outskirts of major populated cities at Egypt (at distances not >40 km from the town centres) were monitored for the presence of pathogenic *E. coli*. These hatcheries were in the Greater Cairo Zone consisting of Cairo Governorate, Giza, Shubra El-Kheima, Helwan, 15^th^ May City, 6^th^ of October City, Shorouk City, Madinaty and Obour City. The total population of this region is estimated at 20,500,000 (as of 2012). The area, density and altitude are 1,709 km^2^, 10,400 people/km^2^ and 250–350 metres (820 and 1,150 ft) above sea level, respectively.

Ethics approval and consent to participate.The meconium samples derived from the birds were all taken from hatchlings in routinely process at the hatchery (H1 thru H10 hatcheries). The hatcheries were numbered in this way, to respect their privacy. No animal experiment was conducted. Meconium samples were collected as fresh droppings on fresh broiler paper at hatcheries. Hand wash samples taken from the workers hands, after their informed and written consent, were part of standard screening by the hatchery officials and these samples became available for the research described in this manuscript. In spite of all these conditions, approval by the Animal Care and Use Committee of the Animal work was approved by the Internal Animal Ethics Committee at Cairo University. Swabs from the environment in the hatcheries and the environment of the broiler farms were taken by the researcher. Sampling was carried out following the procedures of the Animal and Plant Health Agency^42^, formerly Animal Health and Veterinary Laboratories Agency-GOV.UK. Methods and experimental protocols were carried out in accordance with the Helsinki Declaration.

### Recovery and identification of pathogenic *E*. *coli* from environmental and meconium samples

#### Wet sampling and handling

During 2015, the following samples were taken from inside ten commercial hatcheries: 10 meconium drops were pooled into one sample to make a total of 45 meconium samples per hatchery (a total of 450 sampled hatchlings); 24 environmental samples per hatchery from the following sources: water, broken eggshells from each tray, infertile eggs, swabs from the workers hands, air tunnels, floors, incubators, hatchery machines and egg refrigerators. The collection of environmental hatchery samples was made using the following protocol: **s**terile cotton wool moistened swabs (moistened with sterile buffered peptone water, BPW) were used to swab each of the environments from the ten hatcheries. Collected samples were put into sterile plastic bags, and these into boxes, carefully labelled, packed, cooled in an icebox and immediately transported to the laboratory and stored at 4 °C. All samples were immediately processed for microbiological analyses. In order to maximize sample quality, hatchery visits were planned in such a way that they coincided with the day at which fertile eggs were received by the hatchery.

#### Isolation and identification of pathogenic *E. coli*

The isolation procedure adopted for *E*. *coli* identification was outlined in the Food and Drug Administration Bacteriological Analytical Manual (FDA-BAM)^43^. In brief, faeces and liquid samples were inoculated in 225 ml brain heart infusion (BHI) broth at 35 °C for 3 h to facilitate resuscitation of injured cells. These pre-enrichments were then transferred to 225 ml of tryptone phosphate (TP) broth and incubated at 44 °C for 20 h. A volume of enriched broth was then plated onto Levine’s eosin-methylene blue (L-EMB; *E*. *coli* colonies produce a green metallic sheen) and MacConkey (MAC) agar plates and the plates were incubated for 18–24 h at 35 °C. Colonies revealing the characteristic metallic sheen colonies on EMB agar (3–5 colonies from each sample) were subjected to biochemical tests for the identification of *E*. *coli*. Presumptive *E*. *coli* colonies were injected into triple sugar iron and urea agar slants and subjected to the indole test. Colonies exhibiting indole, methyl-red, and catalase tests as positive, Voges–Proskauer and citrate tests as negative, and fermenting glucose and lactose sugars, were confirmed as *E*. *coli* isolates^44^. Colonies showing positive results were further identified by using the API 20E test (bioMérieux Clinical Diagnostics, Marcy l’Étoile, France). *Enterobacter cloacae* American Type Culture Collection (ATCC) 1307 and *E*. *coli* ATCC 11775 were included as positive controls and *Salmonella* Berta ATCC 8392 was included as a negative control.

#### Detection of O-serogroups

Isolates biochemically confirmed as *E*. *coli* in MAC agar were submitted to slide agglutination tests using polyvalent and monovalent sera. Commercial antisera available at the Central Laboratories of Ministry of Public Health, Egypt, were used.

### Phenotypic virulence factors

#### Biofilm formation

Multiple methods (below) of determining biofilm formation, both qualitatively and quantitively, were used to differentiate the propensity of isolate to attach to surfaces and produce biofilms. The Congo red dye assay allows qualitative analysis for biofilm production, the glass test tube assay allows for qualitative analysis of attachment to glass surfaces and subsequence biofilm formation during disturbance and fluid flow, and the microtiter plate method for quantitative analysis of attachment and biofilm formation on plastic surfaces during static conditions.

#### Congo red (CR) dye uptake

The ability to take up Congo red dye was determined on agar plates supplemented with 50 mg/mL of Congo red dye. Five microliters of each bacterial suspension were streaked onto the plates, and the plates were incubated at 37 °C for 24 h. The strong biofilm producing isolates were visualized as black colored colonies with dry crystalline consistency, while the red colonies are interpreted as non-biofilm producer isolates^45^ and moderate biofilm producers were variation between.

#### Glass test tube assay (CTM)

Quantification of biofilms on glass was based on the test tube assay, described by Christensen *et al*.^46^. A loopful of each *E*. *coli* isolate grown overnight in culture plates was inoculated in Brain Heart Infusion (BHI) broth (10 ml), and incubated for 24 h at 37 °C without shaking. The tubes were decanted, washed with PBS (pH 7.3) and air-dried. Dried tubes were stained with crystal violet (0.1%). Excess stain was removed and tubes were washed with deionized water. Tubes were than dried in inverted position and observed for slime layer formation. Biofilm formation was considered positive when a visible film lined the wall and bottom of the tube. Ring formation at the liquid interface was not indicative of biofilm formation. All the isolates were tested in triplicate. Tubes were examined and the amount of biofilm formation was scored as 0-absent, 1-weak, 2-moderate or 3-strong.

#### Microtiter plate assay (MPA)

*E*. *coli* isolates were recovered from −80 °C glycerol stocks onto tryptic soy agar and were stored at 4 °C. For tests in the static biofilm model, and according to the procedure of Wakimoto *et al*.^47^, 200 μL of Dulbecco’s modified Eagle’s medium containing 0.45% glucose in 96-well flat-bottom microtiter polystyrene plates (Becton Dickinson, Franklin Lakes, NJ) were mixed with 5 μl of an *E*. *coli* overnight culture grown at 37 °C in Luria broth. 100 µl from this dilution, were inoculated into eight separate wells of a presterilized polyvinyl chloride (PVC) microtiter plate. Eight wells of Modified Welshimer’s broth were included as a negative control. The plates were incubated for 18 h at 37 °C. After 40 h the liquid was removed from the wells, and unattached cells were removed by rinsing three times with 150 μl of sterile water. Biofilms were stained by adding 50 μl of a 0.5% crystal violet solution to each well and incubating for 45 min at room temperature. Unbound dye was removed by rinsing three times with 150 μl of sterile water. The crystal violet was solubilized by adding 200 μl of 95% ethanol and incubating at 4 °C for 30 min. The contents of each well (100 μl) were then transferred to a sterile polystyrene microtiter plate, and the optical density at 595 nm (OD_595_) of each well was measured in a microplate reader (model 680, Bio rad, USA).

#### Production of hemolysins

The *E*. *coli* hemolytic activity was evaluated by streaking Columbia agar plates (Oxoid) supplemented with 5% sheep blood with each of the isolated *E*. *coli*. After 24 h incubation at 37 °C plates were examined for signs of β-hemolysis (clearing zones around colonies), α-hemolysis (a green-hued zone around colonies) or γ-hemolysis (no halo around colonies)^48^.

### Antibiotic susceptibility testing of the *E*. *coli* isolates

The antimicrobial susceptibility of each *E*. *coli* isolate was tested against a panel of 23 antibiotics (Oxoid) (Table 3), chosen according to their medical importance^49^, by inoculating a calibrated bacterial suspension (0.5 McFarland) on Mueller-Hinton agar. The antibiotic susceptibilities of the isolates were determined using Kirby Bauer’s disk diffusion method as described in the guidelines of the Clinical and Laboratory Standards Institute (CLSI) and the results were interpreted according to CLSI guidelines^50^. *Escherichia coli* ATCC 25922 was used as the quality control strain for the antibiotic susceptibility tests and the results were interpreted as per CLSI criteria.

### Molecular typing methods

#### Detection of *uidA*, ampC and class A β-lactamase genes

*E*. *coli* isolates were grown overnight in 3 ml BHI broth at 37 °C for 18–24 h, after which 200 μl aliquots were transferred to 1.5-ml Eppendorf centrifuge tubes and centrifuged at 13,000 × *g* for 2 min. The bacterial pellets were resuspended in 200 μl of sterile water by vortexing. The suspension was boiled for 10 min and centrifuged for 5 min, after which 150 μl of the supernatant containing DNA was used as the DNA template.

Species identification was confirmed using an *E*. *coli* specific PCR amplification protocol for the *uidA* gene (Table 4)^51^. The optimized protocol was carried out with a PCR mix of 25 *μ*L that contained 2.5 mM MgCl_2_, 20 mM Tris-HCl (pH 9.0 at 25 °C), 50 mM KCl, and 0.1% Triton X-100), 1 mM dNTP mixture, 1 *μ*M of each of the primers, 1 U of Taq polymerase and 1 *μ*L of the DNA template. For the presence of the following resistance genes in the *E*. *coli* isolates: the plasmid-mediated *amp*C genes was investigated using a multiplex PCR assay targeting *bla*_MOX_-like_,_ *bla*_CIT_-like and *bla*_FOX_, as described by Pérez-Pérez and Hanson^52^ and class A β-lactamase genes (*bla*_TEM_, *bla*_SHV_ and *bla*_OXA-1_) as reported by Colom *et al*.^53^ and indicated in Table 4. The PCR reaction was performed with a final volume of 50 µl in 0.5-ml thin-walled tubes. Each reaction contained 20 mM Tris-HCl (pH 8.4); 50 mM KCl; 0.8 mM dNTP mixture; 1.5 mM MgCl_2_; 0.6 µM primers *bla*_MOX_-like and *bla*_CIT_-like; 0.4 µM primers *bla*_FOX_; and 1.25 U of *Taq* DNA polymerase. Template DNA (2 µl) was added to 48 µl of the master mixture. All PCR reactions were overlaid with oil and PCR was performed in a Veriti 96-well thermal cycler. PCR amplicons were then analyzed on a 1.5% agarose gel, stained with ethidium bromide and visualized by Gel Documentation System. A 100 bp DNA ladder (Promega, USA) was used as a size marker.

**Table 4:** PCR-specific oligonucleotide primers, amplicon size and conditions for *uid*A, *amp*C and class A β-lactamase genes.

Name	Target Enzyme(s)	Amplicon Size (bp)	Cycle Number	Annealing Temperature (°C)	Primer Sequence	Conferred Resistance (or Purpose)	Reference
*uidA*	β-glucuronidase specific for *E*. *coli*	486	35	60	P1: 5′-ATCACCGTGGTGACGCATGTCGC	Confirmation of *E*. *coli*	^51^
					P2: 5′-CACCACGATGCCATGTTCATCTGC		
AmpC-like β-lactamase (plasmid associated)							
*bla*_*MOX*_-like	MOX-1, MOX-2, CMY-1, CMY-8 to CMY-11	520	25	64	MOXMF: 5′-GCTGCTCAAGGAGCACAGGAT	Enzymes are known to confer resistance to penicillins, oxyimino group cephalosporins and 7-α-methoxy group. Possible resistance to monobactam aztreonam. Unchanged sensitivity for cefepime and carbapenems^62^.	^52^
					MOXMR: 5′-CACATTGACATAGGTGTGGTGC		
*bla*_*CIT*_-like	LAT-1 to LAT-4, CMY-2 to CMY-7, BIL-1	462	25	64	CITMF: 5′-TGGCCAGAACTGACAGGCAAA		
					CITMR: 5′-TTTCTCCTGAACGTGGCTGGC		
*bla*_*FOX*_	FOX-1 to FOX-5b	190	25	64	FOXMF: 5′-AACATGGGGTATCAGGGAGATG		
					FOXMF: 5′-CAAAGCGCGTAACCGGATTGG		
Extended-spectrum β-lactamase (plasmid associated)							
*bla*_*OXA*-*1*_	OXA-1	609	54	32	OXA-G: 5′-TCAACTTTCAAGATCGCA	ESBL enzymes retain ability to confer resistance to penicillins and confer resistance to expanded-spectrum cephalosporins. Unchanged sensitivity for cephamycins, cefoxitin, and cefotetan^63^.	^53^
					OXA-H: 5′-GTGTGTTTAGAATGGTGA		
*bla*_*SHV*_	SHV	392	54	32	SHV-F: 5′-AGGATTGACTGCCTTTTTG		
					SHV-R: 5′-ATTTGCTGATTTCGCTCG		
*bla*_*TEM*_	TEM	516	54	32	TEM-C: 5′-ATCAGCAATAAACCAGC		
					TEM-H: 5′-CCCCGAAGAACGTTTTC		

### Statistical analysis

Antibiotic resistance phenotypic profiles and gene presence were converted into binary or numerical coding for statistical analysis. Sensitivity to an antibiotic was represented as 0 and resistance was represented as 1. Presence or absence of a specific gene (eg. *bla*_TEM_) was represented as 1 and 0, respectively. Because of the qualitative nature of CTM and Congo red (CR) analysis, results were converted to presence or absence similar to antibiotic resistance profiles. Statistical analysis was performed using the open statistical program R^54^. Correlations for binary variables were calculated using the ‘cor’ function and using ‘cor.test’ function to determine significance by invoking the Pearson method to yield the binary equivalent of the Pearson correlation coefficient, Phi coefficient. Significant correlations were visualized utilizing the ‘corrplot’ function from the ‘corrplot’ R package^55^. A heatmap and hierarchical clustering was performed heatmap3 R package^56^. Distance matrices were calculated for hierarchical clustering analysis by the ‘dist’ function using the ‘binary’ method and clustered by ‘hclust’ using Ward’s method^57^. The R packages ‘FactoMineR’^58^ and ‘factoextra’^59^ were used to calculate and visualize Principal component analysis (PCA). Comparison of serotype impact on CTM and CR was performed using ‘glm’ function with guassian model and followed by post-hoc analysis with ‘glht’ from the ‘MultiComp’ package^60,61^. The impact of serotype on MTP results was statistically determined by use of ‘aov’ function and the post-hoc test ‘TukeyHSD’, if needed. Comparisons between frequencies of occurrence of each phenotypic or genotypic feature in *E*. *coli* isolates from hatchlings or hatchery environments were made by contingency table χ^2^ tests (at *p* < 0.05).

## Electronic supplementary material

Supplementary information
