# Supplementary material for: Poultry hatcheries as potential reservoirs for antimicrobial-resistant Escherichia coli: A risk to public health and food safety
Source: Sci Rep. 2018 Apr 11;8:5859. doi: 10.1038/s41598-018-23962-7 (PMC5895583; doi:10.1038/s41598-018-23962-7)
Supplement: Supplementary file 1 — Supplementary information [file 41598_2018_23962_MOESM1_ESM.docx]

## Poultry hatcheries as potential reservoirs for antimicrobial-resistant *Escherichia coli*: A risk to public health and food safety

**Kamelia M. Osman^1*^,**

^1^Department of Microbiology, Faculty of Veterinary Medicine, Cairo University, Egypt

e-mail: [kamelia-osman@hotmail.com](mailto:kamelia-osman@hotmail.com)

Tel: +20233854762

*Corresponding author

**Anthony D. Kappell^2^,**

^2^Department of Civil, Construction and Environmental Engineering, Marquette University, United States of America;

e-mail: [anthony.kappell@marquettte.edu](mailto:anthony.kappell@marquettte.edu)

**Mohamed Elhadidy^3,4^,**

^3^Department of Bacteriology, Mycology and Immunology, Faculty of Veterinary Medicine, Mansoura University, Mansoura, Egypt;

^4^University of Science and Technology, Zewail City of Science and Technology, Giza, Egypt

e-mail: mmelhadidy@gmail.com

**Fatma ElMougy^5^_,_**

^5^Department of Clinical and Chemical Pathology, Faculty of Medicine, Kasr AlAini, Cairo University, Egypt

e-mail: fatmaelmougy@yahoo.com

**Wafaa A. Abd El-Ghany^6^,**

^6^Department of Poultry Diseases, Faculty of Veterinary Medicine, Cairo University, Egypt

e-mail: wafaa.ghany@yahoo.com

**Ahmed Orabi^1^,**

^1^Department of Microbiology, Faculty of Veterinary Medicine, Cairo University, Egypt

e-mail: drorabi2012@yahoo.com

**Aymen S. Mubarak^7^,**

^7^Department of Botany and Microbiology, College of Science, King Saud University, Kingdom of Saudi Arabia;

e-mail: [aymubarak@ksu.edu.sa](mailto:aymubarak@ksu.edu.sa)

**Turki M. Dawoud^7^,**

^7^Department of Botany and Microbiology, College of Science, King Saud University, Kingdom of Saudi Arabia;

e-mail: [tdawoud@ksu.edu.sa](mailto:tdawoud@ksu.edu.sa)

**Hassan A. Hemeg^8^_,_**

^8^Department of Clinical Laboratory sciences, college of Applied Medical sciences, Taibah University, Saudi Arabia

e-mail: [hasanhemeg@hotmail.com](mailto:hasanhemeg@hotmail.com)

**Ihab M.I. Moussa^7^,**

^7^Department of Botany and Microbiology, College of Science, King Saud University, Kingdom of Saudi Arabia;

e-mail: [imoussa1@ksu.edu.sa](mailto:imoussa1@ksu.edu.sa), Fax: 00966-14678456

Tel.: 0096656749553

**Ashgan M. Hessain^9^,**

^9^Department of Health Science, College of Applied Studies and Community Service, King Saud University, Kingdom of Saudi Arabia;

e-mail: [ahessan@ksu.edu.sa](mailto:ahessan@ksu.edu.sa),Tel.: 00966502646161

**Hend M. Y. Yousef^10^_,_**

^10^Central Administration of Preventive Medicine, General Organization for Veterinary Service, Egypt

e-mail: dr_nody2001@yahoo.com

**Table S1. Antibiotic resistance, hemolytic activity, and biofilm formation ability and antibiotic resistance gene profiles of *E. coli* strains isolated from chicken hatchlings.**

|  |  |  | Antibiotic resistance profile | | | | | | | | | | | | | | | | | | | | | | | Antibiotic resistance genes | | | | | | Hemolysis | | Biofilm formation | | |
| --- | --- | --- | --- | --- | --- | --- | --- | --- | --- | --- | --- | --- | --- | --- | --- | --- | --- | --- | --- | --- | --- | --- | --- | --- | --- | --- | --- | --- | --- | --- | --- | --- | --- | --- | --- | --- |
|  |  |  |  |  |  |  |  |  |  |  |  |  |  |  |  |  |  |  |  |  |  |  |  |  |  |  |  |  |  |  |  |  |  |  |  |  |
|  |  |  |  |  |  |  |  |  |  |  |  |  |  |  |  |  |  |  |  |  |  |  |  |  |  |  |  |  |  |  |  |  |  |  |  |  |
|  |  |  |  |  |  |  |  |  |  |  |  |  |  |  |  |  |  |  |  |  |  |  |  |  |  |  |  |  |  |  |  |  |  |  |  |  |
| Number Isolate | *Serotype* | Hatchery number | Colistin | Cephradine | Fosfomycin | Gentamycin | Chloramphenicol | Neomycin | Enrofloxacin | Ciprofloxacin | Streptomycin | Norfloxacin | Amoxicillin | Ampicillin | Sulfa-Trimethoprim | Doxycycline | Flumequine | Spiramycin | Spectinomycin | Pefloxacin | Rifampicin | Ceftiofur | Oxytetracycline | Clindamycin | Erythromycin | *bla*_TEM_ | *bla*_SHV_ | *bla*_OXA-1_ | *bla*_MOX_-like | *bla*_FOX_ | *bla*_CIT_-like | Beta | Alpha | CR | CTM | MPA (mean OD) |
| 36 | O1:K61 | H4 | S | S | S | S | S | S | R | S | R | R | R | S | S | R | R | R | R | S | R | R | R | R | R | + | - | + | + | - | - | - | + | ++ | strong | 0.1 |
| 37 | O1:K61 | H4 | S | S | S | S | S | S | R | S | R | R | R | R | S | R | R | R | R | R | R | R | R | R | R | + | + | + | + | - | - | - | + | + | moderate | 0.1 |
| 38 | O2:K69 | H6 | S | S | S | S | R | R | R | S | R | R | R | R | R | R | R | R | R | R | R | R | R | R | R | + | - | - | + | - | - | - | + | ++ | weak | 0.2 |
| 39 | O2:K69 | H6 | S | S | S | S | R | R | R | S | R | R | R | R | R | R | R | R | R | R | R | R | R | R | R | + | - | - | + | - | - | - | + | ++ | strong | 0.1 |
| 40 | O8:K60 | H6 | S | S | S | S | R | R | S | S | R | R | R | R | S | R | R | R | R | R | R | S | R | R | R | + | - | - | + | - | + | - | + | + | weak | 0.6 |
| 41 | O8:K60 | H6 | S | S | S | S | R | S | S | R | R | R | R | R | S | R | R | R | R | R | R | S | R | R | R | + | + | - | + | - | + | - | + | ++ | moderate | 0.2 |
| 42 | O8:K60 | H6 | S | S | S | S | R | R | S | S | R | R | R | R | S | R | R | R | R | R | R | S | R | R | R | + | - | - | + | - | + | - | + | +++ | weak | 0.1 |
| 43 | O25:K- | H7 | S | S | S | S | S | S | R | S | S | R | R | R | R | R | R | R | R | R | R | S | R | R | R | + | + | + | - | - | - | - | + | + | weak | 0.1 |
| 44 | O25:K- | H7 | S | S | S | S | S | R | R | S | S | R | R | R | R | R | R | R | R | R | R | S | R | R | R | + | - | + | + | - | - | - | + | + | moderate | 0.1 |
| 45 | O78:K80 | H7 | S | S | S | S | R | R | S | S | R | R | R | R | R | R | R | R | R | R | R | R | R | R | R | + | - | + | + | - | + | - | + | +++ | moderate | 0.2 |
| 46 | O78:K80 | H7 | S | S | S | S | R | R | R | S | R | R | R | R | R | R | R | R | R | R | R | R | R | R | R | + | + | + | + | - | + | - | + | ++ | moderate | 0.1 |
| 47 | O78:K80 | H7 | S | S | S | R | R | S | R | S | R | R | S | R | R | R | R | S | R | S | R | R | R | R | R | + | - | + | + | - | + | - | + | ++ | moderate | 0.1 |
| 48 | O86:K61 | H3 | S | S | S | S | S | R | R | R | R | R | R | R | R | R | R | R | R | R | R | S | R | R | R | + | + | + | + | + | + | - | + | ++ | moderate | 0.2 |
| 49 | O86:K61 | H3 | S | S | S | S | S | R | R | R | R | R | R | R | R | R | R | R | R | R | R | S | R | R | R | + | + | + | + | + | + | - | + | ++ | weak | 0.2 |
| 50 | O86:K61 | H4 | S | S | S | S | S | R | R | R | R | R | R | R | R | R | R | R | R | R | R | S | R | R | R | + | + | + | + | + | + | - | + | ++ | strong | 0.1 |
| 51 | O86:K61 | H4 | S | S | S | S | S | R | R | R | R | R | R | R | R | R | R | S | R | R | R | S | R | R | R | + | + | + | + | + | + | - | + | +++ | moderate | 0.1 |
| 52 | O86:K61 | H2 | S | S | S | S | S | R | S | R | R | R | R | R | R | R | R | R | R | R | R | S | R | S | R | + | + | - | + | + | + | - | + | + | strong | 0.2 |
| 53 | O86:K61 | H2 | S | S | S | S | R | R | R | R | R | R | R | R | R | R | R | R | R | R | R | S | R | R | S | + | + | + | + | + | + | + | - | ++ | strong | 0.1 |
| 54 | O119:k58 | H5 | S | S | S | S | R | R | S | S | R | R | R | R | R | R | R | R | R | R | R | R | R | R | R | + | - | + | + | - | + | - | + | + | moderate | 0.2 |
| 55 | O119:K58 | H5 | S | S | S | S | R | R | R | R | R | R | R | R | R | R | R | R | R | R | S | R | R | R | R | - | - | - | - | - | - | - | + | ++ | strong | 0.1 |
| 56 | O119:K58 | H5 | S | S | S | S | R | R | R | R | R | R | R | R | R | R | R | R | R | R | R | R | R | R | R | - | - | - | - | - | - | - | + | ++ | strong | 0.1 |
| 57 | O128:K71 | H1 | S | S | S | R | R | S | R | S | R | R | S | R | R | R | R | S | R | S | R | R | R | R | R | + | - | + | + | - | + | - | + | + | strong | 0.1 |
| 58 | O128:K71 | H1 | S | S | S | R | R | R | S | S | R | R | S | R | R | R | R | S | R | S | R | R | R | R | R | + | + | + | + | - | + | - | + | + | moderate | 0.1 |
| 59 | O128:K71 | H1 | S | S | S | S | S | S | R | S | R | R | R | S | S | R | R | R | R | S | R | S | R | R | R | + | - | + | + | - | - | - | + | + | moderate | 0.2 |
| 60 | O128:K71 | H1 | S | S | S | S | S | S | R | S | R | R | R | S | S | R | R | R | R | S | R | S | R | R | R | + | + | + | + | - | - | - | + | ++ | moderate | 0.1 |
| 61 | O158:K | H2 | S | S | S | S | S | R | S | S | R | R | R | R | R | R | S | R | R | R | S | R | R | R | R | + | - | + | + | - | + | - | + | ++ | weak | 0.1 |
| 62 | O158:K | H2 | S | S | S | S | S | R | S | S | R | R | R | R | R | R | S | R | R | R | S | R | R | R | R | + | - | - | + | - | + | - | + | ++ | moderate | 0.1 |
| 63 | O158:K | H3 | S | S | S | S | S | R | S | S | R | R | R | R | R | R | S | R | R | R | S | R | R | R | R | + | - | - | + | - | + | - | + | ++ | strong | 0.2 |
| 64 | O164:K- | H3 | S | S | S | S | R | R | S | S | R | S | R | R | R | R | R | R | R | R | R | R | R | R | R | + | - | - | + | + | + | - | + | + | strong | 0.1 |
| 65 | O164:K- | H4 | S | S | S | S | R | R | S | S | R | S | R | R | R | R | R | R | R | R | R | R | R | R | R | + | - | - | + | + | + | - | + | +` | moderate | 0.2 |

R, resistant; **S**, susceptible (Zone diameter based on CLSI standards)

CR, Congo red agar; CTM, Christensen’s Tube Method; MPA, Microtiter plate assay

OD: optical density; OD_595_ < 0.1 = weak; 0.1 ≤ OD_595_ ≤ 1.0 = strong; OD_595_ > 1 = very strong

**Table S2. Phenotypic (antibiotic resistance, hemolytic activity and biofilm formation ability) and genotypic (antibiotic resistance genes) profiles of all confirmed *E. coli* strains isolated from the hatchery environment.**

|  |  |  |  | Antibiotic resistance profile | | | | | | | | | | | | | | | | | | | | | | | Antibiotic resistance genes | | | | | | Hemolysis | | Biofilm formation | | |
| --- | --- | --- | --- | --- | --- | --- | --- | --- | --- | --- | --- | --- | --- | --- | --- | --- | --- | --- | --- | --- | --- | --- | --- | --- | --- | --- | --- | --- | --- | --- | --- | --- | --- | --- | --- | --- | --- |
| Isolate | Serotype | Hatchery number | Environment | Colistin | Cephradine | Fosfomycin | Gentamycin | Chloramphenicol | Neomycin | Enrofloxacin | Ciprofloxacin | Streptomycin | Norfloxacin | Amoxicillin | Ampicillin | Sulfa-Trimethoprim | Doxycycline | Flumequine | Spiramycin | Spectinomycin | Pefloxacin | Rifampicin | Ceftiofur | Oxytetracycline | Clindamycin | Erythromycin | *bla*_TEM_ | *bla*_SHV_ | *bla*_OXA-1_ | *bla*_MOX_-like | *bla*_FOX_ | *bla*_CIT_-like | Beta | Alpha | CR | CTM | MPA (mean OD) |
|  |  |  |  |  |  |  |  |  |  |  |  |  |  |  |  |  |  |  |  |  |  |  |  |  |  |  |  |  |  |  |  |  |  |  |  |  |  |
| 1 | O25:K11 | H4 | Air tunnel | S | S | S | S | S | R | R | S | R | S | R | R | R | R | R | R | R | R | R | R | R | R | R | + | - | - | - | - | - | - | + | +++ | moderate | 0.2 |
| 2 | O25:K11 | H4 | Incubators | S | S | S | S | S | R | R | S | R | S | R | R | R | R | R | R | R | R | R | R | R | R | R | + | - | + | + | - | - | - | + | +++ | weak | 0.1 |
| 3 | O25:K11 | H1 | Workers’ hands | S | S | S | S | S | S | R | S | R | R | R | R | R | R | R | R | R | R | R | R | R | R | R | + | + | + | - | - | - | - | + | ++ | strong | 0.2 |
| 4 | O25:K11 | H6 | Hatchery machines | S | S | S | S | S | R | R | S | R | R | R | R | R | R | R | R | R | R | R | R | R | R | R | + | - | + | - | - | - | - | + | ++ | moderate | 0.1 |
| 5 | O44:K74 | H7 | Air tunnel | S | S | S | S | S | R | R | R | R | R | R | R | R | R | R | R | R | R | R | S | R | R | R | + | - | + | - | - | - | - | + | + | moderate | 0.1 |
| 6 | O44:K74 | H2 | Infertile egg | S | S | S | S | S | R | R | R | R | R | R | R | S | R | R | R | R | R | R | S | R | R | R | + | + | + | - | - | - | - | + | ++ | weak | 0.1 |
| 7 | O44:K74 | H7 | Infertile egg | S | S | S | S | S | R | S | S | R | R | R | R | R | R | R | R | R | R | R | S | R | R | R | + | - | + | - | - | - | - | + | + | moderate | 0.2 |
| 8 | O44:K74 | H7 | Hatchery machines | S | S | S | S | S | R | R | R | R | R | R | R | R | R | S | R | R | R | S | R | R | R | R | + | - | + | - | - | - | - | + | ++ | moderate | 0.2 |
| 9 | O44:K74 | H2 | Water | S | S | S | S | S | R | S | R | R | R | R | R | R | R | S | R | R | R | S | R | R | R | R | + | - | - | - | - | + | - | + | ++ | weak | 0.1 |
| 10 | O44:K74 | H2 | Egg refrigerators | S | S | S | S | S | R | R | R | R | R | R | R | R | R | R | R | R | R | S | R | R | R | R | + | - | - | - | - | - | - | + | ++ | moderate | 0.1 |
| 11 | O78:K80 | H3 | Incubators | S | S | S | S | S | R | R | R | R | S | S | S | R | R | R | R | R | R | S | R | R | R | R | + | - | + | - | - | - | - | + | +++ | moderate | 0.1 |
| 12 | O78:K80 | H4 | Infertile egg | R | S | S | S | R | S | S | R | R | S | R | R | R | R | R | R | R | R | R | R | R | R | R | + | - | + | - | - | - | - | + | +++ | moderate | 0.2 |
| 13 | O78:K80 | H4 | Floor | S | S | S | S | S | R | R | S | S | S | R | R | R | R | R | R | R | R | R | R | R | R | R | + | + | + | - | - | - | - | + | ++ | moderate | 0.2 |
| 14 | O78:K80 | H4 | Egg refrigerators | S | S | S | S | S | S | R | S | R | R | R | S | S | R | R | R | R | S | R | R | R | R | R | + | - | + | - | - | - | - | + | +++ | strong | 0.1 |
| 15 | O114:K90 | H6 | Air tunnel | S | S | S | S | R | R | R | R | R | R | R | R | R | R | R | R | R | R | R | S | R | R | R | + | - | + | - | - | - | - | + | ++ | weak | 0.2 |
| 16 | O114:K90 | H3 | Incubators | S | S | S | S | R | R | R | R | R | R | R | R | R | R | R | R | R | R | R | S | R | R | R | + | - | + | - | - | - | - | + | ++ | strong | 0.2 |
| 17 | O114:K90 | H6 | Incubators | S | S | S | S | R | R | R | R | R | R | R | R | R | R | R | R | R | R | R | S | R | R | R | + | - | + | - | - | - | - | + | ++ | weak | 0.2 |
| 18 | O114:K90 | H6 | Infertile egg | S | S | S | S | R | R | R | R | R | R | R | R | R | R | R | S | R | R | R | S | R | R | R | + | - | + | - | - | + | - | + | +++ | moderate | 0.1 |
| 19 | O114:K90 | H4 | Hatchery machines | S | S | S | S | S | R | S | R | R | R | R | R | R | R | R | R | R | R | R | S | R | S | R | + | - | - | - | - | - | - | + | ++ | weak | 0.1 |
| 20 | O114:K90 | H2 | Water | S | S | S | S | R | R | R | R | R | R | R | R | R | R | R | R | R | R | R | S | R | R | S | - | + | + | - | - | - | + | - | ++ | strong | 0.1 |
| 21 | O119:K69 | H3 | Incubators | S | S | S | S | R | S | S | R | S | R | R | R | R | S | R | R | R | R | R | R | R | R | R | + | - | + | - | - | - | - | + | ++ | strong | 0.1 |
| 22 | O119:K69 | H2 | Workers’ hands | R | S | S | S | R | S | R | R | R | S | R | R | R | R | R | S | R | R | R | R | R | R | R | + | - | + | + | - | - | - | + | +++ | strong | 0.1 |
| 23 | O119:K69 | H3 | Hatchery machines | S | S | S | S | R | R | S | S | R | S | R | R | R | R | R | S | R | R | R | R | R | R | R | + | - | + | - | - | - | - | + | +++ | moderate | 0.2 |
| 24 | O119:K69 | H3 | Water | S | S | S | S | R | R | S | S | R | R | R | R | R | R | R | R | R | R | R | R | R | R | R | + | - | + | - | - | - | - | + | ++ | moderate | 0.1 |
| 25 | O119:K69 | H2 | Floor | S | S | S | S | R | R | R | S | R | R | R | R | R | R | R | R | R | R | R | R | R | R | R | + | - | + | - | - | - | - | + | ++ | moderate | 0.1 |
| 26 | O126:K71 | H7 | Incubators | S | S | S | S | S | R | S | S | R | S | R | R | R | R | S | R | R | S | R | S | R | R | R | + | - | + | + | - | - | + | - | +++ | moderate | 0.1 |
| 27 | O126:K71 | H7 | Infertile egg | S | S | S | S | S | S | R | S | S | R | R | R | R | R | S | R | R | R | R | R | R | R | R | + | - | + | - | - | + | - | + | ++ | strong | 0.1 |
| 28 | O126:K71 | H5 | Workers’ hands | S | S | S | S | S | S | R | S | R | R | R | R | S | R | R | R | R | R | R | R | R | R | R | + | - | + | - | - | - | - | + | ++ | strong | 0.2 |
| 29 | O126:K71 | H3 | Hatchery machines | S | S | S | S | R | R | R | R | R | R | R | R | R | R | R | R | R | R | S | R | R | R | R | + | - | + | - | - | - | - | + | + | moderate | 0.1 |
| 30 | O126:K71 | H6 | Floor | S | S | S | S | R | R | R | R | R | R | R | R | R | R | R | R | R | R | R | R | R | R | R | + | - | + | - | - | - | - | + | ++ | moderate | 0.1 |
| 31 | O126:K71 | H6 | Egg refrigerators | S | S | S | S | S | R | R | R | R | R | S | R | R | R | R | R | R | R | R | R | R | R | R | + | - | + | - | - | - | - | + | ++ | moderate | 0.2 |
| 32 | O128:K71 | H5 | Incubators | S | S | S | S | S | R | R | R | R | R | R | S | R | R | R | R | R | R | R | R | R | R | R | + | + | + | - | - | - | - | + | +++ | moderate | 0.2 |
| 33 | O128:K71 | H1 | Infertile egg | S | S | S | S | R | R | R | R | R | R | S | R | R | R | R | R | R | R | R | R | S | R | R | + | - | + | + | - | - | - | + | +++ | moderate | 0.1 |
| 34 | O128:K71 | H1 | Infertile egg | S | S | S | S | R | R | R | R | R | R | R | R | R | R | S | R | R | R | R | R | R | R | R | + | - | + | - | - | - | + | - | ++ | strong | 0.1 |
| 35 | O128:K71 | H5 | Water | S | S | S | S | S | R | S | R | R | R | R | R | R | R | R | R | R | R | R | R | R | R | R | + | - | + | - | - | - | - | + | ++ | strong | 0.1 |

R, resistant; **S**, susceptible (Zone diameter based on CLSI standards)

CR, Congo red agar; CTM, Christensen’s Tube Method; MPA, Microtiter plate assay

OD: optical density; OD_595_ < 0.1 = weak; 0.1 ≤ OD_595_ ≤ 1.0 = strong; OD_595_ > 1 = very strong
